# Connection between high pore-fluid pressure and frictional instability at tsunamigenic plate boundary fault of 2011 Tohoku-Oki earthquake

**DOI:** 10.1038/s41598-022-16578-5

**Published:** 2022-08-08

**Authors:** Ehsan Jamali Hondori, Jin-Oh Park

**Affiliations:** 1 Atmosphere and Ocean Research Institute, The University of Tokyo, Kashiwa, Japan; 2 Present Address: Geoscience Enterprise Inc. (GSE), Tokyo, Japan

**Keywords:** Geophysics, Seismology, Tectonics

## Abstract

The 2011 Tohoku-Oki earthquake (M 9.0) rupture propagated along a shallow plate boundary thrust fault (i.e. decollement) to the trench, displaced the seafloor, and triggered a devastating tsunami. Physical properties of the underthrust sediments which control the rupture propagation are yet poorly known. We use a 2D seismic dataset to build velocity model for imaging and apply reverse time migration. We then calculate pore-fluid pressure along the decollement as the top boundary of underthrust sediments, and along the backstop interface as the boundary between undeformed structures in the continental plate and the severely deformed sediments in the accretionary prism. The results show that within horizontal distance of 40–22 km toward the trench, pore-fluid pressure is 82–60% higher than the hydrostatic pressure for both decollement and backstop interface. It then reduces to hydrostatic level for the backstop interface but remains 60–40% higher than hydrostatic level for the decollement, causing frictional instability in favor of fault rupture along the decollement. We report for the first time, by our knowledge, detailed seismic images of fluid-rich trapped bucket sediments, quantitative stress states, and fluid drainage conditions at shallow tsunamigenic portion of the Japan Trench, which are consistent with the seafloor and borehole observations.

## Introduction

Large coseismic slip was previously considered unlikely to happen in the shallow parts of the subduction megathrust faults^1,2^, where overlying less-consolidated sediments could presumably deform aseismically. However, the 2011 Tohoku-Oki earthquake (M 9.0) with a large coseismic slip^3–6^ of more than 50 m raised a critical issue regarding the process which could enable the full rupture propagation along the plate boundary thrust fault (i.e., decollement) up to the trench. Laboratory friction experiments on samples retrieved from Integrated Ocean Drilling Program (IODP) Japan Trench Fast Drilling Project (JFAST) site C0019 showed a very low shear stress at the shallow plate boundary due to the presence of smectite-rich pelagic clay and thermal pressurization of the pore-fluids^7^. Borehole temperature observatory data also obtained a very low apparent friction coefficient of 0.08 by analyzing the dissipated frictional heat at the plate boundary^8^.

Despite the invaluable findings of the JFAST experiments, the pin-point drilling site may reflect local features which are, to some extent, expandable in the along-strike direction only. Moreover, there is no information about the spatial distribution of pore-fluid pressure crucial to understand earthquake fault slip behavior. Physical properties of the underthrust sediments substantially control the pore-fluid pressure and stress distribution^9^. Tectonic loading, rapid burial, and mineral dehydration of the subducting sediments can increase the pore-fluid pressure, which results in reduced vertical effective stress and shear stress at the plate boundary^10^. Therefore, it is necessary to quantitatively explore downdip variation of the physical properties in a direction normal to the trench axis. Several studies have used seismic reflection data and empirical relationships to evaluate P-wave velocity (V_p_), porosity, pore-fluid pressure, and effective stress in different subduction zones such as Nankai Trough^10,11^, southern Ecuador^12^, Cascadia^13^, Alaska^14^, and Hikurangi^9^.

**Figure 1:**
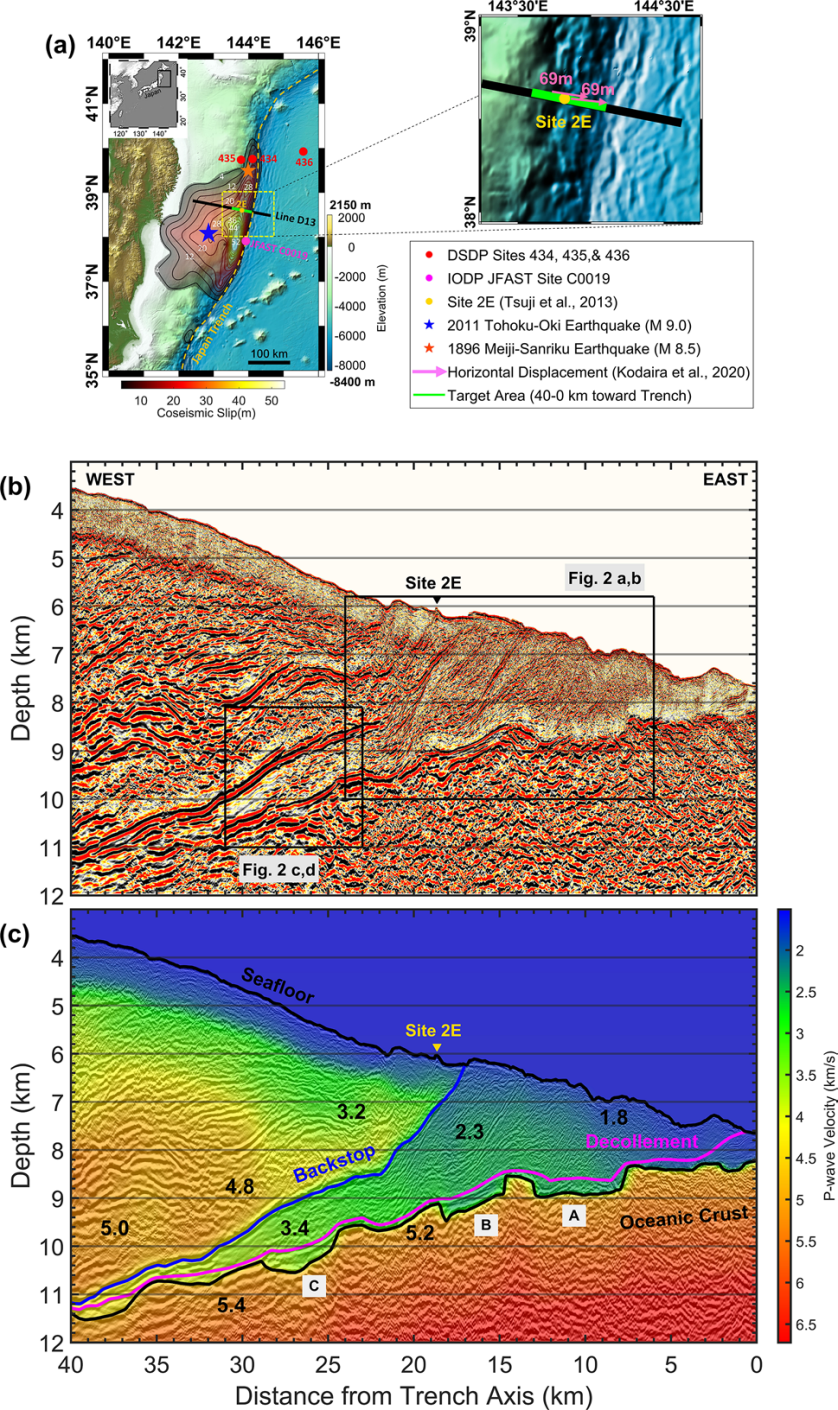
Study area in the Japan Trench. (**a**) Survey map of the 2D seismic line D13 (black line), target area within 40–0 km distance toward the trench (green line), site 2E from previous seafloor observations^15^ (yellow circle), epicenter of the 2011 Tohoku-Oki earthquake (blue star), the 2011 coseismic slip calculated by tsunami waveform inversion^5^ (color-shaded contour lines), epicenter of the 1896 Meiji Sanriku tsunami earthquake (orange star), Deep Sea Drilling Project (DSDP) sites 434, 435, and 436 (red circles), and IODP JFAST drilling site C0019 (magenta circle). A zoomed rectangular view around the target area shows the location of 69 m seafloor displacements (magenta arrows) due to the 2011 Tohoku-Oki earthquake^17^. (**b**) RTM section of line D13 within a range of 40–0 km distance. (**c**) P-wave velocity model and major horizon interpretations overlaid on the RTM image of line D13. Reflection from backstop interface (blue line) sets the boundary between deformed sediments in the wedge-shaped low-velocity zone and undeformed structures of the upper plate. Top of the oceanic crust (black line) marks the sharp basement of the horst-and-graben structure, and the decollement (magenta line) indicates the plate boundary fault. Grabens marked with capital letters A, B, and C are discussed in the main text. Numbers printed in black across the image are representative V_p_ values in km/s.

Here, we develop a reliable V_p_ model and apply RTM (“Methods” section) on 2D seismic reflection data from line D13 (Fig. 1) to obtain high-fidelity seismic depth images. We then calculate pore-fluid pressure, vertical effective stress, and shear stress along the decollement and backstop interface within a horizontal distance of 40–0 km toward the trench. It should be noted that all the horizontal distances in this paper are implicitly referred to the trench axis, but we do not mention it anymore to avoid redundancy. We carefully assess the uncertainty of the results at each step and report the first quantitative in-situ stress state derived from active-source seismic reflection data acquired in the Japan Trench subduction zone offshore Miyagi, northeast Japan. Our results match very well with previous seafloor observations^15^, differential bathymetry studies^6,16,17^, and JFAST drilling site C0019 experiments^7,8^ to provide a reliable tool for evaluating the stress pattern in a direction normal to the trench axis.

## Results

### Characteristic structures on RTM depth section

The Pacific plate subducts beneath the Okhotsk plate at a convergence rate of 8.6 cm/year^18^ in the Japan Trench margin. Previous geophysical and geological studies^19–21^ showed that while in the southern part of Japan Trench an elongated channel-like sedimentary unit is being subducted, in the northern part a small accretionary prism is formed. This wedge-shaped low-velocity zone is separated from the continental framework by backstop interface, a landward-dipping strong reflector which sets the boundary between the deformed sediments (Vp of 2.0–4.0 km/s) in the accretionary prism and undeformed structures (V_p_ of 3.0–5.0 km/s) in the upper plate (Fig. 1b,c). The incoming oceanic plate is characterized by a horst-and-graben pattern, which originated at the bending-related normal faults^22^. Core samples retrieved from IODP JFAST site C0019 at ~ 7 km distance from the Trench showed that the plate boundary decollement is localized onto an interval of smectite-rich pelagic clay, and steeply dipping pelagic and/or hemipelagic mudstone are accreted above the highly fractured plate boundary^23,24^.

**Figure 2:**
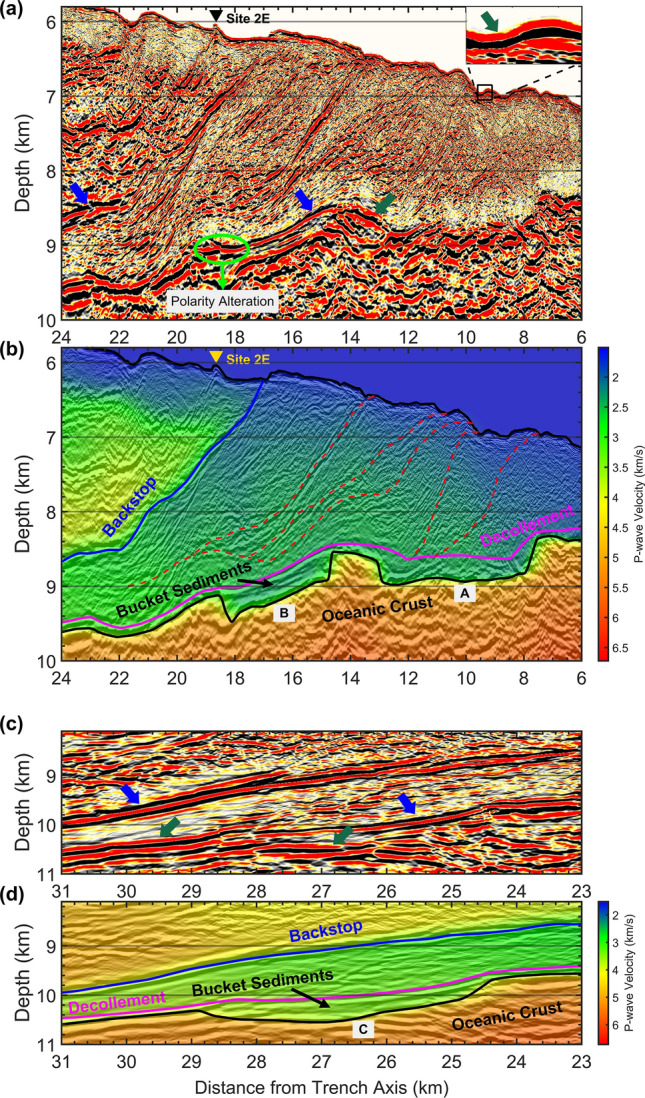
Depth imaging of bucket sediments. (**a**,**c**) RTM section and (**b**,**d**) P-wave velocity model overlaid on RTM image for two zoomed panels indicated by black rectangles in Fig. 1b. The dark green arrows point to the reflections with a normal polarity same as the seafloor reflection, as indicated by the inset in panel (**a**), while the blue arrows point to the reflections with a reverse polarity. Decollement reflection has a very week amplitude at graben A, but shows a high amplitude with reverse polarity at grabens B and C. Some landward dipping thrust faults (red dashed lines) reach the plate boundary at graben A and help the pore-fluids to be drained out from this graben. However, similar thrust faults do not touch the plate boundary at Graben B or C and pore-fluids are maintained there to make a strong seismic reflectivity. The lateral alteration in the polarity marked by green oval in panel (a) indicates the boundary between reflection from bucket sediments and reflection from top of the basaltic oceanic crust.

Our V_p_ model shows the wedge-shaped low-velocity accretionary prism and underthrust sediments (Fig. 1b,c; see Figs. S1, S2 in the supplementary material for the whole profile). The RTM depth image (Fig. 2a,c) shows the reflection from backstop interface with a polarity opposite to the seafloor reflection. A steep cliff which was previously observed^15^ by manned submersible Shinkai 6500 at site 2E has been well imaged by RTM (see Fig. S3 in the supplementary material for a comparison with Kirchhoff pre-stack depth migration). The grabens in the oceanic plate provide accommodation space for the fluid-rich pelagic sediments to be carried into the greater depths by a relatively rapid subduction. Decollement reflection shows a very weak amplitude at graben A, where a series of small-scale landward dipping thrust faults have developed across the plate boundary (dashed red lines in Fig. 2b). Decollement reflection with a high amplitude and reverse polarity at top of grabens B and C imply that the pore-fluids are maintained in the trapped sediments, here after called as *bucket sediments*, resulting in an even lower seismic velocity within these grabens. Although the limited resolution of the tomographic approach did not allow us to capture this velocity reduction, the reverse polarity of the decollement reflection provides a good measure for the interpretation of these bucket sediments (Fig. 2b,d).

### Physical properties of the sediments

**Figure 3:**
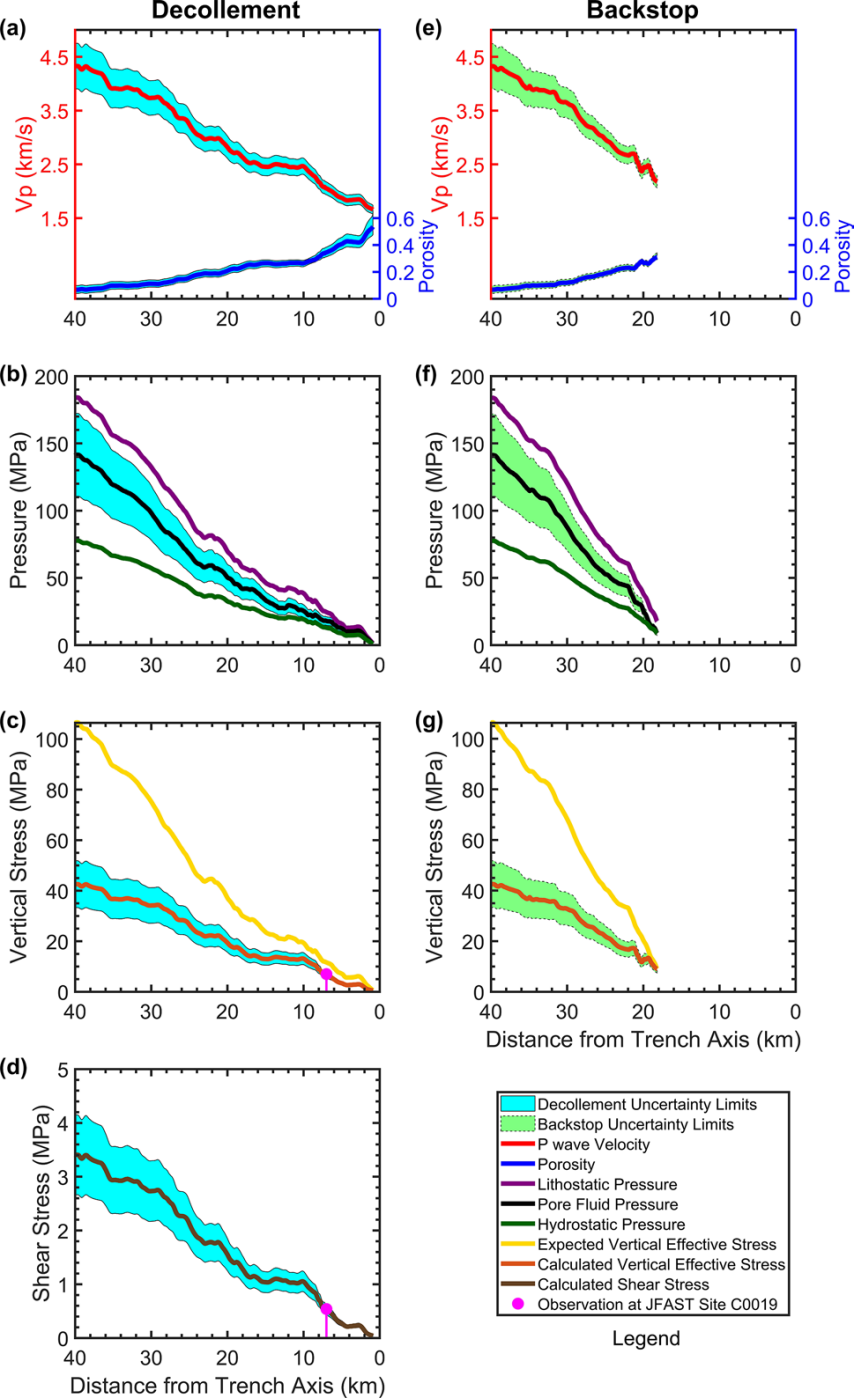
Physical properties calculated for the decollement (left column) and backstop interface (right column). (**a**,**e**) P-wave velocity (red line) extracted at the corresponding horizon depth and the associated porosity values (blue line) transformed by an empirical relationship (“Methods” section). (**b**,**f**) Lithostatic (purple line), hydrostatic (dark green line), and pore-fluid (black line) pressure values. (**c**,**g**) Calculated vertical effective stress (maroon line) and expected vertical effective stress under normal consolidation conditions (yellow line). (**d**) Shear stress calculated at decollement level. The magenta filled circles in panels (**c**) and (**d**) mark the previous borehole observation results^7,8^ from JFAST site C0019. The uncertainty bands indicated in cyan (for decollement) and light green (for backstop) include the cumulative errors of velocity to porosity transformation, consolidation curve fitting, and V_p_ inaccuracies (“Methods” section).

The extracted V_p_ values increase downdip for both backstop interface and decollement, but still are considered low for their corresponding burial depths (Fig. 3a,e). Pore-fluid pressure is approximately 82–60% higher than the hydrostatic pressure for both decollement and backstop within 40–22 km horizontal distance. The pore-fluid pressure of the backstop then quickly reduces to hydrostatic level within 22–18 km but remains 60–40% higher than hydrostatic pressure for the decollement within 22–2 km (Fig. 3b,f). The vertical effective stress for decollement and backstop interface at 40 km distance is almost 43 MPa but the expected vertical effective stress at this location is 106 MPa, which is 146% larger. The uncertainty limits (“Methods” section) reach to a maximum of 22% at 40 km distance and are considered negligible compared to 146% of difference between the calculated in-situ and expected vertical effective stress values (Fig. 3c,g). Since line D13 and JFAST drilling site C0019 are both located within the large coseismic slip area of 2011 Tohoku-Oki earthquake (Fig. 1a) and are only 70 km away from each other, we use the observations made at JFAST site C0019 to evaluate the reliability of our results. A previous study^8^ at JFAST drilling site C0019 considered a vertical effective stress of 7 MPa and analyzed the temperature anomaly at the plate boundary, caused by the dissipated frictional heat due to the 2011 Tohoku-Oki earthquake, to estimate an apparent coefficient of friction of 0.08 and slip-averaged shear stress of 0.54 MPa. We multiplied a coefficient of friction of 0.08 to our vertical effective stress values and calculated the shear stress along the decollement (Fig. 3d), assuming that cohesion is negligible^25^. Considering that JFAST site C0019 is ~ 7 km away from the trench^23^, our resulting vertical effective stress of 6.588 MPa and shear stress of 0.527 MPa at 7 km horizontal distance from the trench are in very good agreement with JFAST borehole observations (Fig. 3c,d). It should be noted that the JFAST borehole temperature observatory was installed 16 months after the 2011 Tohoku-Oki earthquake and it observed the data for 9 months before the complete sensor string was recovered^8^. So, those observations are in fact made with a considerable time lag after the 2011 Tohoku-Oki earthquake. The seismic data of line D13 was acquired in May 2011, only two months after the earthquake. We believe this seismic data provides an insight which is much closer to the coseismic physical state of the plate boundary fault.

### Fluid drainage conditions

**Figure 4:**
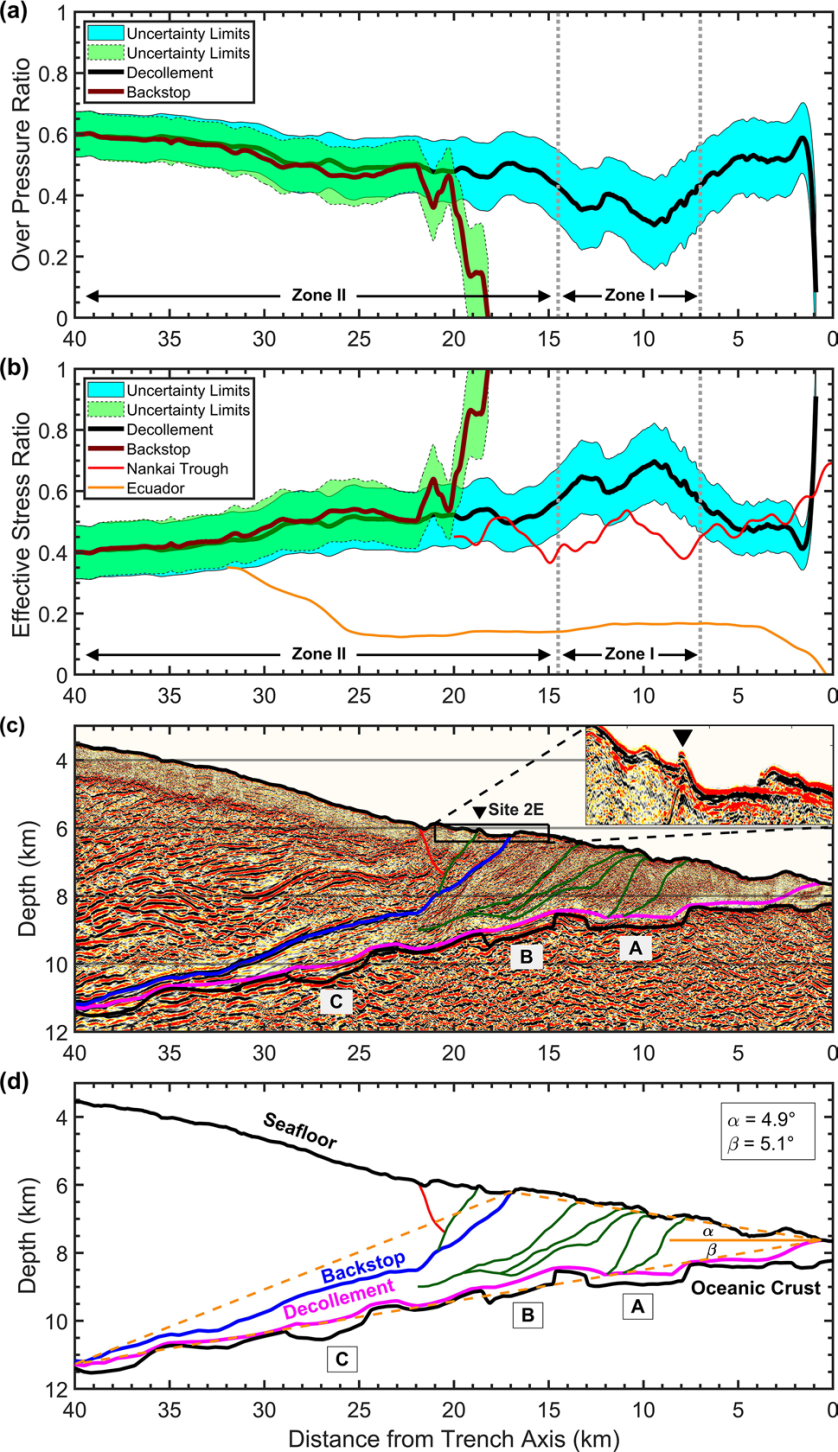
Fluid drainage, stress state, and fault strength. (**a**) Pore-fluid overpressure ratio for decollement (black line) and backstop interface (maroon line) shows that backstop interface is well drained within a distance of 22–18 km due to the active drainage system through a reverse fault reaching to the seafloor at site 2E. Two zones are identified across the decollement, Zone I has a better drainage for the fluid expulsion but Zone II has a poor drainage condition with fluids mostly maintained in the pore spaces. (**b**) Effective stress ratio along the decollement (black line), backstop interface (maroon line), Nankai Trough^11^ (red line), and Southern Ecuador^12^ (orange line). Larger values of effective stress ratio imply on stronger fault coupling while lower values indicate that fault is mechanically weak and prone to slip. For Japan Trench, backstop interface is stronger than decollement within 22–18 km distance and is not a preferable path for fault slip to the seafloor. The effective stress ratio of decollement is lower than 1.0 along the whole profile, despite the higher values in Zone I, which result in frictional instability and rupture propagation to the seafloor. (**c**) Interpreted reverse faults (dark green lines) and a normal fault (red line) on the RTM image. (**d**) Schematic drawing of the wedge zone and seismic interpretations. A triangle shown by dashed orange lines is used to define surface slope angle α and basal dip β to validate our results by calculating effective coefficient of basal friction (μ_b_′) using an independent method of critical taper theory.

We calculated fluid overpressure ratio (λ*, “Methods” section) to evaluate the pore-fluid drainage conditions. If the subsurface materials are fully undrained and the fluids are maintained in the pore spaces (i.e., the media is impermeable) then a maximum overpressure ratio of 1.0 will be obtained. On the other hand, an overpressure ratio of 0.0 indicates optimal drainage conditions for the pore-fluid evacuation (i.e., the media is totally permeable)^12^. In line D13, the overpressure ratio along backstop interface and decollement is approximately 0.6–0.5 within 40–22 km distance. At the backstop interface, overpressure ratio rapidly reduces to 0.0 within 22–18 km distance (Fig. 4a), indicating that pore pressure reaches to hydrostatic level and an active drainage system is developed there. This area is beneath site 2E, where a previous seafloor observation^15^ by submersible Shinkai 6500 detected Calyptogena at a steep cliff (Fig. 4c), suggesting that seepage has occurred along the reverse fault linking backstop interface to the seafloor at site 2E (green line above backstop in Fig. 4c,d). The biological colonies found there require warm water for living and this is an evidence that the reverse fault reaching to site 2E from backstop has provided the path for the pore-fluid expulsion, which explains why the pore-fluid pressure at backstop interface within this zone is lower. A normal fault (red line in Fig. 4c,d) may also contribute to the drainage of the pore-fluids. It should be noted that due to the very thin thickness of the sediments above backstop interface between horizontal distance of 18–17 km, this area has been excluded from our analysis.

We identify two different zones based on the variations in overpressure ratio along the decollement. Zone I with a low overpressure ratio between 0.3 and 0.45 spans over 7–14.5 km horizontal distance, where excess pore pressure is 5–10 MPa and pore-fluids support 65%–73% of the overburden stress (Fig. 4a). The RTM section shows that landward-dipping small-scale fractures and thrust faults reach to the plate boundary at graben A in this zone and develop a drainage path for the pore-fluids to be expelled out from the underthrust sediments. On the other hand, Zone II is located within a horizontal distance of 14.5–40 km with a landward increasing overpressure ratio of 0.45–0.60. The excess pore pressure in Zone II ranges between 10 and 64 MPa and pore-fluids support 73%–77% of the overburden stress. Although a number of reverse faults have developed in this zone (Fig. 4c,d), those faults do not touch the plate boundary. The opposite polarity of reflection from bucket sediments in grabens B and C indicates that pore-fluids are preserved there due to a poor drainage condition. The landward increase in the overpressure ratio could be due to the rapid tectonic loading and fast burial associated with forearc thickening, which does not keep pace with the fluid expulsion from the underthrust sediments.

In order to further investigate the reliability of the results, we followed an independent approach of critical taper theory^26,27^ to calculate the effective coefficient of basal friction (μ_b_′) by using pore-fluid pressure ratio (λ), surface slope angle α, and basal dip β (Fig. 4d). The details of this method are out of scope of this paper and the interested reader is referred to the references^26,27^ for further information. The average pore-fluid pressure ratio in our results for the decollement is 0.73 within a distance of 40–2 km. Using this value we obtained an effective coefficient of basal friction (μ_b_′) equal to 0.09 for the plate boundary fault beneath the wedge-shaped low-velocity zone along line D13. An apparent coefficient of friction of 0.08 had been previously estimated from borehole temperature observatory data at JFAST site C0019 by calculating the dissipated frictional heat during the earthquake from the observed temperature anomaly at the plate boundary fault^8^. The effective coefficient of basal friction obtained by the critical taper theory represents the static frictional resistance of the plate boundary fault, whereas the apparent coefficient of friction estimated from the dissipated frictional heat represents the dynamic frictional resistance during the earthquake. A different study showed that the static coefficient of friction at the plate boundary fault beneath the Japan Trench forearc is almost same as the dynamic coefficient of friction estimated from frictional heating analysis^27^. The nearly same static and dynamic coefficient of friction could possibly imply in two facts. First, the fluid-rich underthrusting sediments with a dominant clay lithology have a dramatically low static coefficient of friction^24,28^. Second, the plate boundary fault has not healed yet by the time of the seismic data acquisition and a long enough period of time is needed until the frictional state of the fault is stabilized^29^. It should be noted that the seismic data of line D13 was acquired just two months after the 2011 Tohoku-Oki earthquake, whereas the JFAST C0019 borehole observations were made more than one year after the earthquake. We believe our results are close to the coseismic physical state of the plate boundary fault. An effective coefficient of basal friction of 0.09 represents a compressively critical state^27^ of the plate boundary fault beneath the low-velocity wedge. This is consistent with the fold-and-thrust structures imaged within the sediments^30^ and the reverse faults interpreted on the RTM section (Fig. 4c).

## Discussion

Backstop interface approaches decollement in the downdip direction and it is quite difficult to distinguish differences between these two boundaries just by looking at the vertical effective stress values (Fig. 3c,g). To evaluate fault strength and slip behavior, here we introduce a parameter as “*effective stress ratio”*, defined by the ratio of the calculated vertical effective stress to the expected vertical effective stress under normal consolidation conditions. Normal consolidation condition means if the sediments were deposited and consolidated without being subject to additional stress caused by the subduction, i.e. if there was no underthrusting. Under normal consolidation conditions, the pore-fluid pressure should be at hydrostatic level and the expected effective stress is calculated by subtracting the hydrostatic pore-fluid pressure from the overburden (lithostatic) pressure. Higher effective stress ratios imply on a stronger fault coupling while lower values denote that the fault is weakly coupled and is prone to slip. The effective stress ratio is approximately 0.4–0.55 for the backstop interface within a distance of 40–22 km then increases steeply to reach a maximum value of 1.0 within 22–18 km distance, denoting that the fault coupling becomes stronger in this area (Fig. 4b). The RTM image (Fig. 4c) confirms that the backstop fault geometry has changed at a horizontal location of 22 km and depth of 8.5 km, from a gently landward dipping reflector with dip angle of 9° to a steep shape with dip angle of 25°. This fault geometry had a direct impact on strengthening the backstop interface against shallow coseismic slip during the 2011 Tohoku-Oki earthquake, as the decollement provided a preferentially flatter interface for the rupture propagation^31^. Seafloor observations at site 2E after the Tohoku-Oki earthquake^15^ did not show any clear deformation or open fissures associated with the earthquake, although such features were observed at several locations^15^ almost 50 km south of line D13. Differential bathymetry studies^6,16,17^ showed that seafloor experienced a horizontal displacement of 69 m at both sides of backstop seafloor exposure (Fig. 1a) and the overriding plate moved seawards mostly coherently. These observations are consistent with the resulted effective stress ratio, showing that backstop interface was not a preferable path for the major fault slip during the Tohoku-Oki earthquake.

At the decollement level, although Zone I has a relatively higher effective stress ratio of 0.55–0.7, Zone II shows a landward decreasing effective stress ratio of 0.55–0.40 (Fig. 4b) implying on a weakly-coupled plate boundary fault. The low effective stress ratio at the whole decollement influences fault slip behavior in two ways. First, it facilitates occurrence of slow slip and very low frequency events during interseismic periods of megathrust earthquakes. Second, it affects the shallow coseismic slip and rupture propagation pattern leading to overshoot during the meguthrust earthquake. A number of transient slow slip events occurred before the 2011 Tohoku-Oki earthquake^32–34^. Previous laboratory shear experiments on samples from JFAST drilling site C0019 showed that the sheared samples have a full spectrum of fault slip, from fast unstable slip to slow steady creep, and the entire shallow plate boundary from 15 km depth to the trench axis is capable of generating slow slip events^35^. The slip at very shallow depths shows the ability of up-dip strain accumulation and tsunamigenic potential of the megathrust^36^. We believe the low effective stress ratio along the whole decollement is a key factor which facilitates occurrence of the slow slip events. Additionally, the local fluctuations in the effective stress ratio, which reflect non-uniform in-situ stress states along the decollement, result in a heterogenous slip behavior. The mechanically weak decollement with a low effective stress ratio enabled the large shallow coseismic slip and full rupture propagation to the trench, resulting in the unexpectedly huge tsunami. The 1896 Meiji Sanriku tsunami earthquake had a coseismic slip area overlapping with the Tohoku-Oki earthquake^37^ (Fig. 1a), and there is a possibility that the low effective stress ratio played a key role in that event, too.

According to a previous study^3^, the Tohoku-Oki earthquake had a dynamic and complex rupture pattern including a small initial phase, a deep rupture for maximum 40 s, a large shallow rupture at 60 s to 70 s, and a continuous deep rupture for more than 100 s. A dynamic rupture simulation to examine the role of stress concentration and thermal fluid pressurization on the rupture process of 2011 Tohoku-Oki earthquake^38^ showed that the plate boundary fault first ruptured the near hypocenter area with a large slip of the order of 15 m. Then, an efficient dynamic thermal pressurization of the pore-fluids helped the slow rupture to propagate eastward and reach the trench to cause an extremely large slip near the trench. The large slip in the near-trench area propagated a secondary rupture over the plate boundary fault in the opposite direction^38^. The large shallow slip near the trench and reversal of the rupture propagation direction occurred due to dynamic overshoot, which is caused by shear stress reduction below dynamic friction^3^. We believe after the nucleation of the 2011 Tohoku-Oki earthquake with an updip rupture propagation from deep hypocenter, the pore-fluids at the mechanically weak near-trench shallowest part of the plate boundary fault were significantly over-pressured. The extremely low effective stress at the shallow plate boundary fault near the trench, due to very high pore-fluid pressure at the decollement level, facilitated the shear stress reduction which eventually led to overshooting and additional rupture in the reverse direction.

We also estimated effective stress ratio for Nankai Trough^11^ (red line in Fig. 4b) and southern Ecuador^12^ (orange line in Fig. 4b) by referring to the previous studies. Similar to Japan Trench, previously reported very low frequency earthquakes in Nankai Trough^39,40^ are distributed over the spatial location of the low effective stress ratio. The effective stress ratio of southern Ecuador subduction channel is extremely low within a horizontal distance of 0–25 km, then starts to increase downdip (Fig. 4b). A previous study^41^ showed that the near-trench part of the southern Ecuador subduction channel continuously creeps with no record of large seismicity, while subduction earthquakes occur at relatively large focal depths much farther from the trench. These are consistent with our effective stress ratio analysis, emphasizing the role of this ratio in controlling the fault strength and slip behavior in different subduction zones.

It should be mentioned that, the pore-fluid pressure and stress state have been previously well studied for different subduction zones and there is always a tradeoff between the available data and the uncertainty in the results^9–14^. For example, when deriving an empirical relationship between V_p_ and porosity the core samples are key factors in constraining the best fit. Similarly, thick incoming sedimentary units help to extract a consolidation curve which spans over a wide range of stress values. Despite the limitations our data may have, as any other dataset in the real-world would, we took several quality control measures to examine the reliability of the results against direct seafloor observations at site 2E, differential bathymetry data, JFAST borehole experiments, and the independent critical taper theory. Nevertheless, we confirmed that the maximum uncertainty of the results remains within a reasonable range. Any possible source of the data in the future, for example additional IODP core samples, may help to reduce the uncertainty and achieve a higher accuracy. Moreover, our future work is to apply the same methodology to additional seismic lines and cover a larger area in the Japan Trench subduction zone.

## Methods

### Seismic data acquisition and processing

Shortly after the 2011 Tohoku-Oki earthquake (M 9.0), Japan Agency for Marine-Earth Science and Technology (JAMSTEC) operated 2D seismic reflection survey KR11-E03 by using research vessel Kairei in May 2011 to investigate the deep crustal structure of Japan Trench subduction zone offshore Miyagi. A large volume tuned airgun array was used as seismic source with a 50 m shot interval and 10 m towing depth. Data was recorded for 18 s at each shot point using a 444-channel streamer cable with 12.5 m group spacing towed at a depth of 21 m. The data were acquired along several 2D lines, some of which were previously used^21^ to characterize the structural variations in the Tohoku-Oki earthquake rupture zone. Here we use data acquired along line D13 (Fig. 1) to calculate the physical properties of the sediments. The spatial location of this line coincides with 2D seismic line MY101, which was acquired in 1999, and several studies^6,15–17,30^ have been conducted to estimate the coseismic slip of 2011 Tohoku-Oki earthquake around this line. Previously, we used line D13 in a different study^42^ to apply seismic full waveform inversion on the shallow sedimentary strata in the forearc upper slope.

We processed the seismic data to prepare it for depth imaging by applying bubble suppression, deghosting, swell noise removal, surface related multiple elimination (SRME), residual multiple suppression using parabolic radon transform (PRT), time-variant band-pass filtering, and FX deconvolution. We developed an initial P-wave interval velocity model by using the layer stripping approach^42^ and further updated the velocity model using several iterations of grid-based traveltime tomography^43^. For the sake of computational efficiency, we applied Kirchhoff prestack depth migration^44^ (KPSDM) during the velocity model building stage but after obtaining the final velocity model we applied reverse time migration^45–47^ (RTM) to produce the seismic depth sections with the highest quality from the target area within 40–0 km distance toward the trench. Because RTM does not require any high-frequency approximation, has no dip restrictions, and can properly image multipath events the RTM images have a better quality and higher accuracy than KPSDM images (see Fig. S3 in the supplementary material for a comparison between RTM and KPSDM images). The final P-wave velocity model (see Figs. S4–S9 in supplementary material for uncertainty analysis of V_p_ model) showed that a wedge-shaped low-velocity zone extends downdip from trench axis landward to a horizontal distance of 40 km. The trench axis is located at the central point of a graben with the deepest seafloor reflection and the decollement horizon starts at the deformation front, which is almost 1.0 km away from the trench axis^48^.

### Estimation of physical properties and their uncertainties

We use an empirical relationship^49^ to calculate porosity from P-wave velocity. This relationship was originally developed by laboratory experiments using core samples from Nankai Trough, however, we noticed that porosity values estimated using this relationship (Fig. S10 in supplementary material) are also in good agreement with the core samples retrieved from IODP JFAST site C0019, Deep Sea Drilling Project (DSDP) sites 434, 435, 436, and Shimanto complex laboratory experiments^50^. The converted porosity values are used to estimate void ratio and bulk density, assuming a grain density of 2.59 g/cm^3^ and water density of 1.024 g/cm^3^. We derive a logarithmic-linear relationship between the hydrostatic vertical effective stress and void ratio^51^ for the incoming sediments, where pore pressure is assumed to be hydrostatic (Fig. S11 in supplementary material). The logarithmic-linear curve fitting approach has been widely used as a standard method for deriving a consolidation trend from the incoming sediments^11,14^. One of the controlling factors here is the thickness of the incoming sediments, as thicker sediments provide a wider range of stress values and improve the accuracy of the fitted consolidation curve. Therefore, we have applied several quality control measures on our results (“Discussion” section) to make sure that the fitted consolidation curve is reliable. The resulting consolidation curve is used to transform porosity to in-situ vertical effective stress along the decollement horizon and backstop interface. We also calculate lithostatic pressure, which is caused by the weight of overlying sediments, including the weight of pore-fluids, by integrating the bulk density values from seafloor down to the target horizon (i.e., decollement or backstop interface) level and multiplying the result by the gravitational acceleration. The pore-fluid pressure is then calculated by subtracting the in-situ vertical effective stress from lithostatic pressure. Dividing the difference between pore-fluid pressure and hydrostatic pressure by the difference between lithostatic pressure and hydrostatic pressure will yield the fluid overpressure ratio (λ*), a useful parameter to evaluate the fluid drainage conditions^9,12^. Pore-fluid pressure ratio (λ) is defined as the ratio between pore-fluid pressure and lithostatic pressure and is used as a key factor in estimating the effective coefficient of basal friction using critical taper theory^26,27^.

We followed an analogy stating that the maximum total uncertainty is accumulated by the uncertainties in Vp model, velocity to porosity transform, and void ratio to effective stress transform^10,11^. We consider an uncertainty of 5% in the P-wave velocity of incoming undeformed sediments. We also assume that uncertainty of P-wave velocity within seafloor to decollement depth laterally increases from 5% in the vicinity of the trench axis, to 10% at a horizontal location 40 km away from the trench axis. This velocity uncertainty is generally lower for backstop interface due to a shallower depth, but since backstop reaches the decollement in the downdip part of the section, we consider a similar trend (i.e., 5%–10%) for the velocity uncertainty at the backstop interface. The average uncertainty of transforming P-wave velocity into porosity is almost 3.5%, which is estimated by normalized standard error between the predicted porosities from the empirical relationship^49^ and observed values from core samples of JFAST site C0019, DSDP sites 434, 435, 436, and Shimanto Complex^50^ (Fig. S10 in supplementary material). The maximum propagated uncertainty, caused by V_p_ model and velocity to porosity transformation, is 8.5% for the incoming sediments approaching to the trench axis and it increases to 13.5% for the underthrust sediments of the decollement level at a location 40 km away from the trench axis. A logarithmic-linear curve fitting approach is used to derive the consolidation trend of the incoming sediments (Fig. S11 in supplementary material) with a normalized RMS error of almost 17% between the fitted curve and data points. If we exclude the 8.5% propagated uncertainty of the incoming sediments velocity and porosity, we obtain an 8.5% uncertainty solely associated with the void ratio to effective stress transformation. In other words, the maximum total uncertainty in the stress calculation is obtained by summation of the uncertainty in void ratio to effective stress transformation (8.5%) and propagated uncertainty of the underthrust sediments velocity and porosity at 0–40 km distance from the trench axis (8.5%–13.5%). To summarize, the maximum total uncertainty in the resulting stress values is 17% for the deformation front at 1.0 km distance from the trench axis and it increases to 22% for a horizontal location 40 km away from the trench axis. We use the same maximum total uncertainty ranges of 17–22% for the backstop interface between a distance of 18–40 km, which sets slightly higher bounds on the real values but confines the error properly. Variations in the bulk density may also introduce uncertainty in the resulting stress values, however, empirical relationships between bulk density and P wave velocity^52^ showed that bulk density is directly related with the 4th root of the velocity. So, the error caused by bulk density variation is negligible compared to uncertainty propagated by velocity model. In addition, the consolidation curve derived from incoming sediments may not fully accommodate the consolidation behavior of the sediments at backstop interface, and it may introduce a possible small amount of uncertainty in the results. Finally, we conducted an error analysis test by intentionally decreasing the slope of the consolidation curve (Fig. S11 in supplementary material) by 10% to see how much the vertical effective stress values would change. This perturbation shifted the vertical effective stress of the decollement from the original value of 6.588 MPa to 8.123 MPa for a horizontal distance of 7 km from the trench axis. Obviously this resulting vertical effective stress is a suboptimal solution due to the increased RMS error, and is largely different from the previously reported^8^ vertical effective stress of 7 MPa at JFAST site C0019 for the same distance from the trench axis. Therefore, we believe the current logarithmic-linear consolidation curve is the best fit using the available data.

## Supplementary Information

Supplementary Information 1.

## Data Availability

The 2D seismic data used in this research can be requested from the JAMSTEC database repository (https://www.jamstec.go.jp/obsmcs_db/e/survey/data_year.html?cruise=KR11-E03). The bathymetry data, shown in the maps, is obtained from GEBCO Compilation Group (2020), GEBCO 2020 Grid (10.5285/a29c5465-b138-234d-e053-6c86abc040b9) https://www.gebco.net/data_and_products/gridded_bathymetry_data/. The coseismic slip data is provided by Satake et al. (2013).

## Code availability

The RTM code for seismic imaging is developed by the corresponding author and is maintained by the author.
