# Supplementary material for: Connection between high pore-fluid pressure and frictional instability at tsunamigenic plate boundary fault of 2011 Tohoku-Oki earthquake
Source: Sci Rep. 2022 Aug 8;12:12556. doi: 10.1038/s41598-022-16578-5 (PMC9360034; doi:10.1038/s41598-022-16578-5)
Supplement: Supplementary file 1 — Supplementary Information 1. [file 41598_2022_16578_MOESM1_ESM.pdf]

Supplementary Material for

Connection between high pore-fluid pressure and frictional instability at tsunamigenic plate boundary fault of 2011 Tohoku-Oki earthquake

Ehsan Jamali Hondori[1*] and Jin-Oh Park[1]

[1]Atmosphere and Ocean Research Institute, The University of Tokyo, Kashiwa, Japan.

ehsan.jamali@aori.u-tokyo.ac.jp

jopark@aori.u-tokyo.ac.jp

Contents of this file

Seismic Depth Sections and Velocity Model

Velocity Uncertainty Analysis

Fitting a Consolidation Curve

Figures S1 to S11

Introduction

Here we illustrate the result of Kirchhoff prestack depth migration (KPSDM) imaging of the full 2D seismic data along line D13 together with the P-wave velocity model. We also describe the procedure which has been used to estimate uncertainty in P-wave velocity model. Moreover, we provide the details of a curve fitting approach used for the consolidation curve estimation from the incoming sediments. Figs. S1-S11 are also provided for additional clarifications.

Seismic Depth Sections and Velocity Model

The Pacific plate is subducting beneath Okhotsk plate in the Japan Trench subduction zone. The 2D seismic data along line D13 could image the major geological features as the KPSDM section in Fig. S1 shows. The P-wave velocity model, which is developed using a layer stripping method and several iterations of grid-based traveltime tomography, is shown in Fig. S2. The black rectangle marks the target area where a wedge-shaped low-velocity zone between backstop interface and oceanic crust is found. We focus on this area, located at 40-0 km horizontal distance toward the trench axis to estimate pore fluid pressure and effective stress to investigate the plate boundary fault slip behavior. For the sake of computational efficiency, we applied reverse time migration (RTM) only on this target area. Fig. S3 shows a comparison between KPSDM and RTM on imaging steeply dipping structures in the dashed rectangle from Fig. S1, where KPSDM could not resolve the reflectors correctly but RTM images the subsurface features properly.

Velocity Uncertainty Analysis

In order to assess the accuracy of our P-wave velocity model, we perturbed the interval velocity values below seafloor by various percentages and compared the flatness, continuity, and resolution of reflections in the resulting KPSDM common image gathers (CIG) at several locations marked as S4-S9 with vertical dashed lines in Figs. S1, S2. The uncertainty in the velocity of the incoming sediments is less than 5%. The velocity uncertainty for a horizontal location at 10 km distance from the trench axis is 5% at 9 km depth, while the uncertainty increases to 10 % at a depth of 12 km for a location 52 km away from the trench axis, as shown in Figs. S4-S9. A green arrow points to the reflection from the top of basaltic oceanic crust as a reference.

Fitting a Consolidation Curve

First, we confirm that the empirical relationship between P wave velocity and porosity[1] is valid for the Japan Trench seismic data. Fig. S10 shows that porosity values estimated using this relationship are in good agreement with the core samples retrieved from IODP JFAST site C0019, DSDP sites 434, 435, 436, and Shimanto complex laboratory experiments[2]. Then we can convert the P-wave velocity of the incoming sediments to porosity and calculate the void ratio. Based on the porosity values from JFAST core samples we exclude the void ratio values exceeding 1.5, which correspond to a maximum porosity of 0.6. We derive a logarithmic-linear relationship between the hydrostatic vertical effective stress and void ratio[3] for the incoming sediments, where pore pressure is assumed to be hydrostatic. The resulting logarithmic-linear consolidation curve for our data is defined by the following equation:

$$e = -0.5605 \log(\sigma_v') + 0.9858,$$

where, e denotes void ratio and vertical effective stress of the incoming sediments (in MPa) is shown by σ_v'. Fig. S11(a) shows the fitted logarithmic-linear function overlaid on the void ratio and hydrostatic vertical effective stress data. For a better visualization, only 2% of the data points have been displayed in this panel. Fig. S11(b) and (c) show a 3D histogram of the whole data distribution density and a planar top-view of the 3D histogram, respectively, to check the closeness of the fitted logarithmic-linear curve to the densely located data points. The histogram is calculated by binning the void ratio and hydrostatic effective stress values over an equally-spaced 150x150 grid and number of data points in each bin are shown by the columns. Note that displaying the hydrostatic effective stress in logarithmic scale exaggerates the horizontal dimensions of the bins. As the planar top-view of the 3D histogram shows, the fitted consolidation curve

(red line) crosses through the area with highest data distribution density. Here we use Root Mean Square Error (RMSE) to evaluate the goodness of the fit of the logarithmic-linear function. The RMSE of 17% was obtained for the best fitted line as in the above equation.

References

1. Hoffman, N. W., and Tobin, H. J. An empirical relationship between velocity and porosity for underthrust sediments in the Nankai Trough accretionary prism, *Proc. Ocean Drill. Program Sci. Results* 190/196, 1–23 (2004).
2. Tsuji, T. et al. Modern and ancient seismogenic out-of-sequence thrusts in the Nankai accretionary prism: Comparison of laboratory-derived physical properties and seismic reflection data. *Geophys. Res. Lett.* **33**, L18309 (2006).
3. Saffer, D. Pore pressure development and progressive dewatering in underthrust sediments at the Costa Rican subduction margin: Comparison with northern Barbados and Nankai, *J. Geophys. Res.* **108**, 2261 (2003).

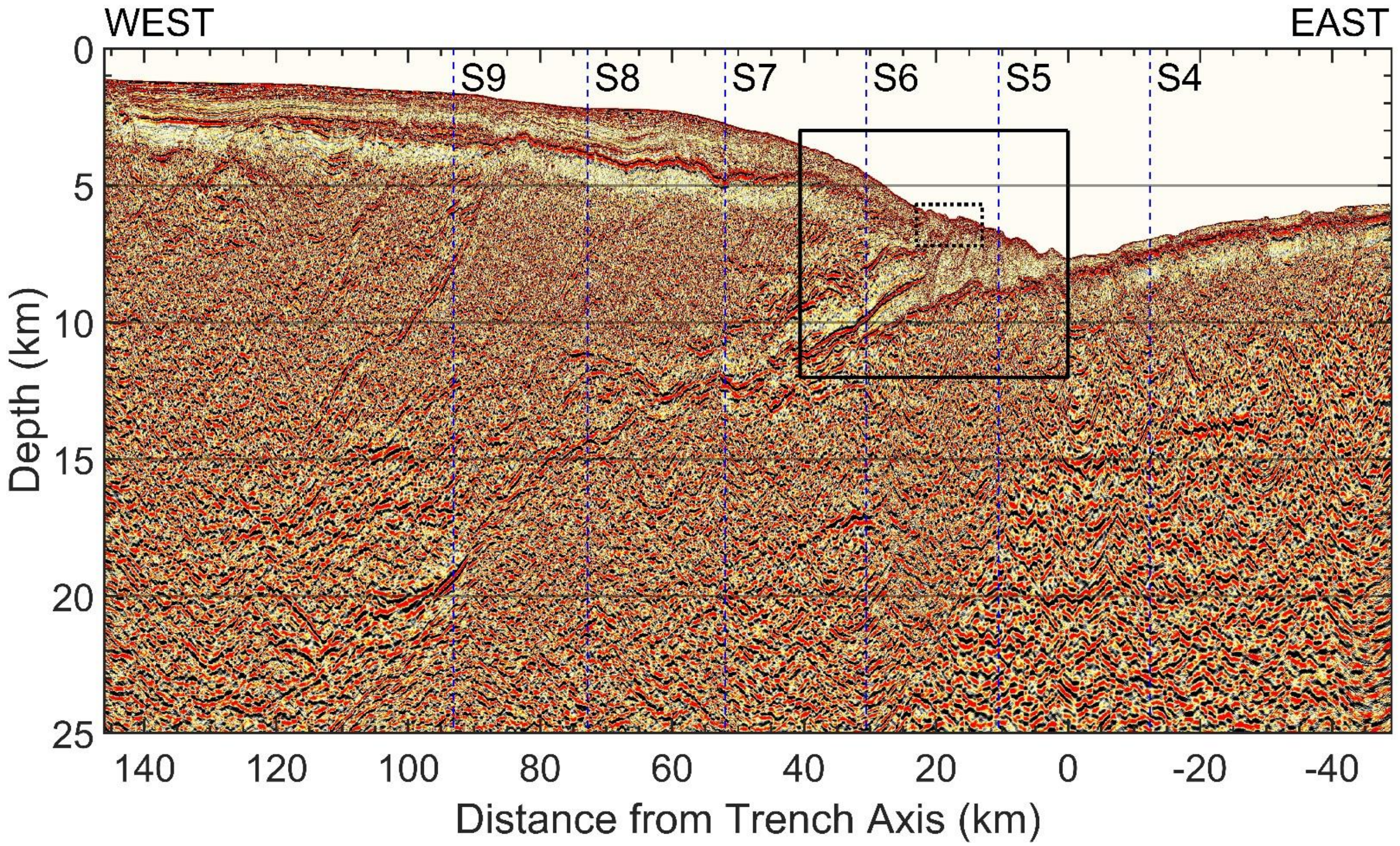

Fig. S1 | KPSDM image of the 2D seismic line D13. Black rectangle marks the target area with a wedge-shaped low velocity zone within 40-0 km to trench. Dashed small rectangle shows the area for comparing KPSDM and RTM in Fig. S3. The vertical dashed lines labeled with S4-S9 indicate the locations for the velocity uncertainty analysis panels shown in Figs. S4-S9.

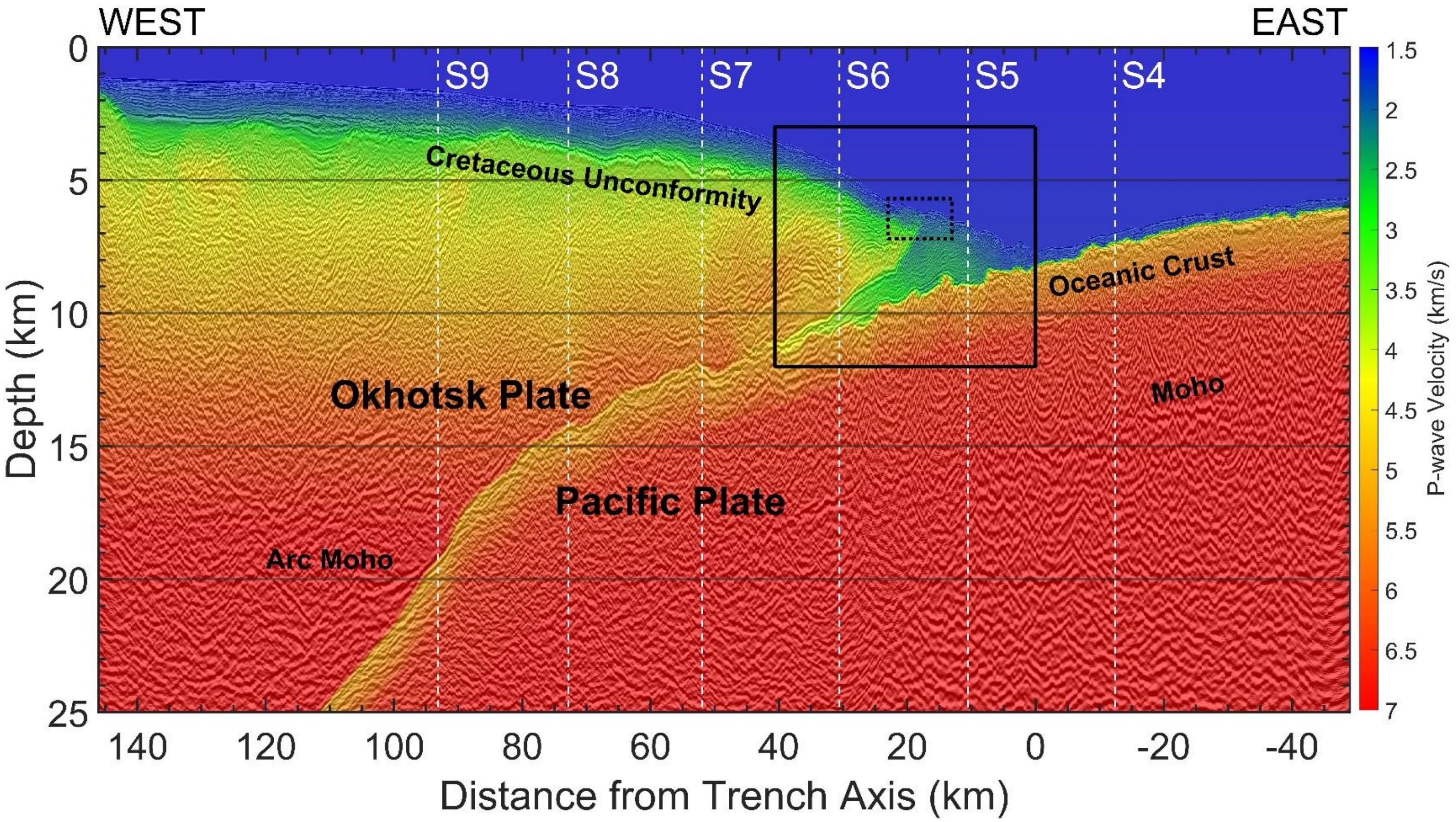

Fig. S2 | P-wave Velocity Model overlaid on KPSDM image of the 2D seismic line D13. The wedge-shape low-velocity zone within the black rectangle is the target area in the main text. Reflections from cretaceous unconformity, oceanic Moho, and arc Moho are also indicated with the labels.

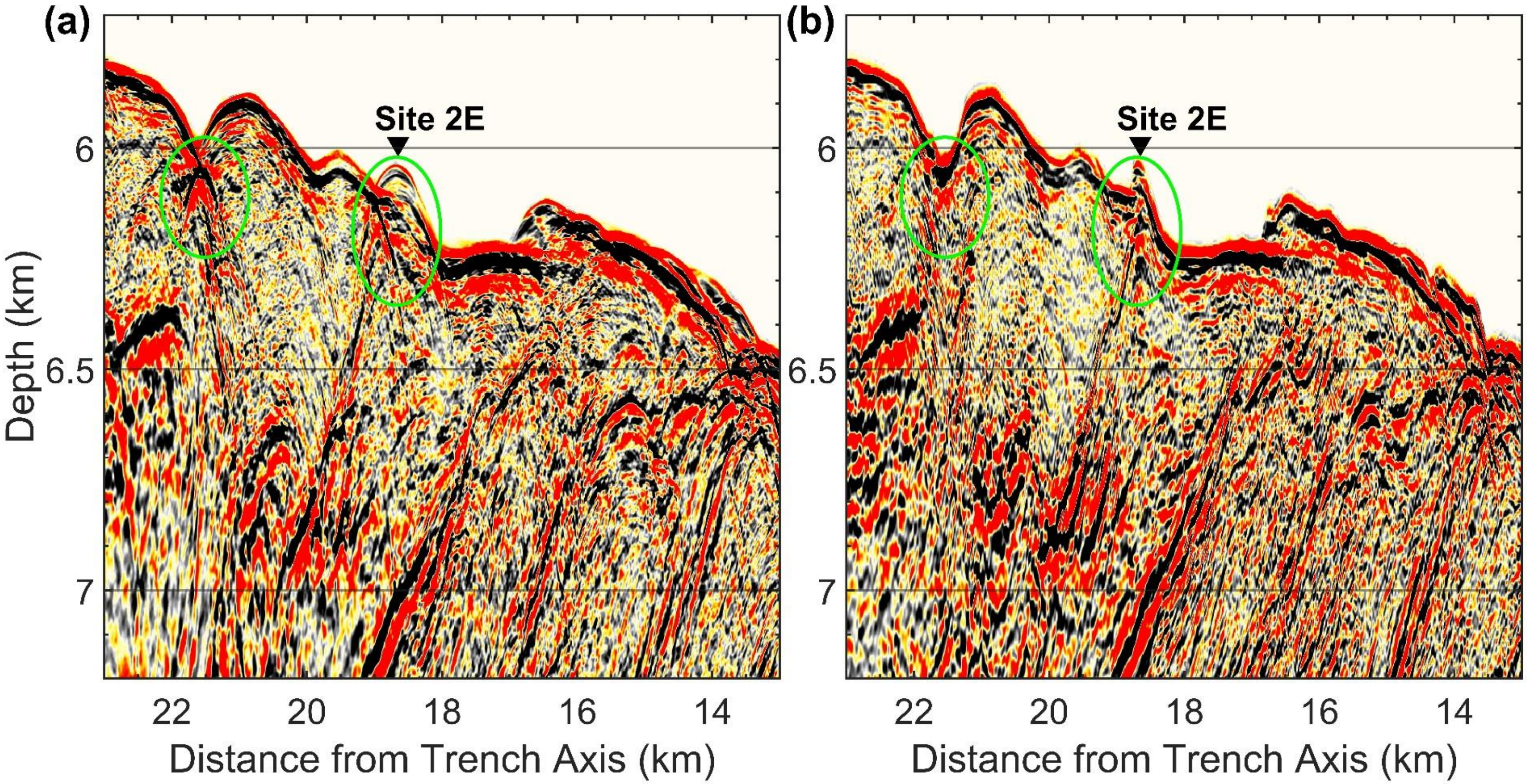

Fig. S3 | Comparison between KPSDM and RTM images. (a) KPSDM section and **(b)** RTM section. Note the area marked by green ovals, where RTM better resolves the steeply dipping reflectors.

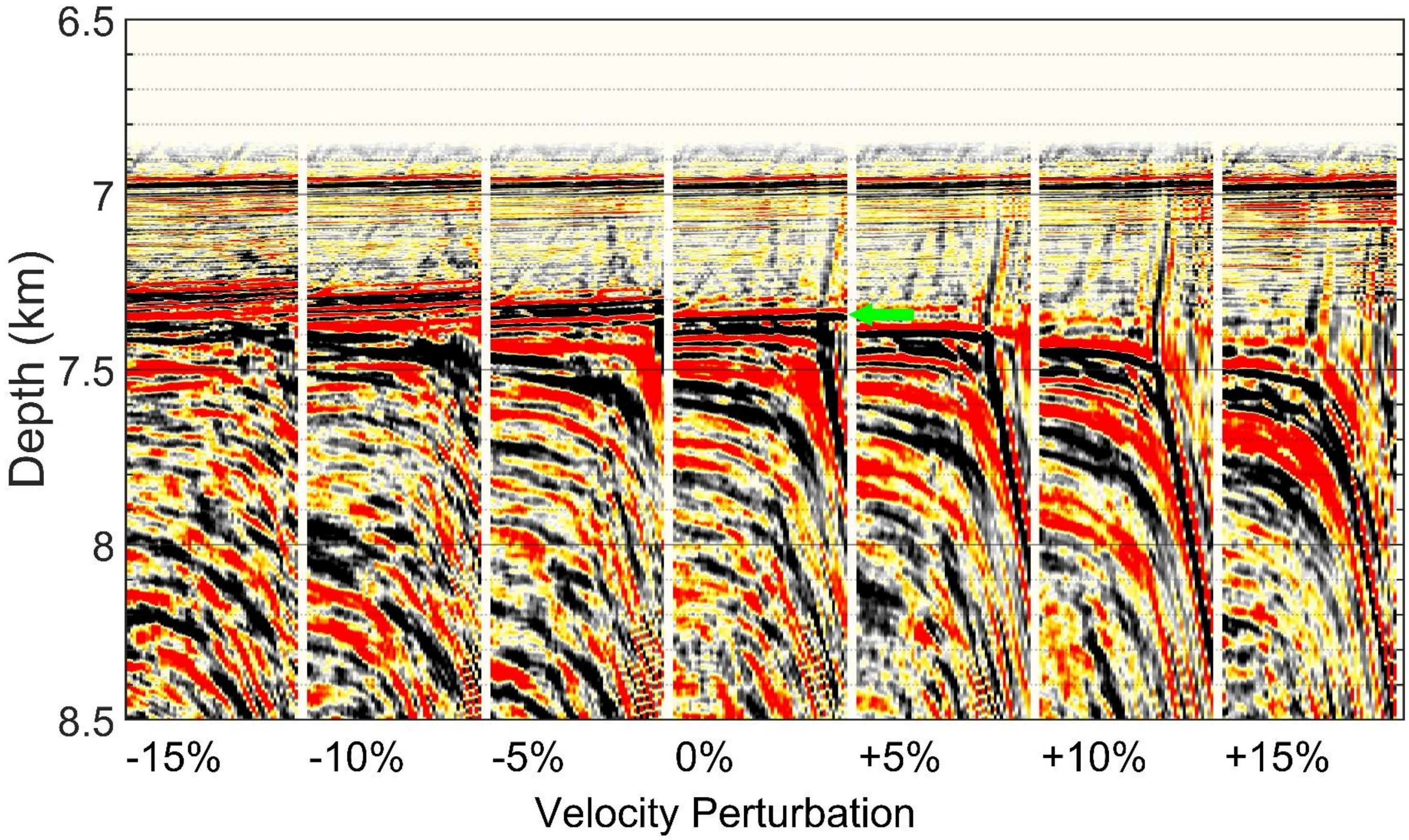

Fig. S4 | Velocity uncertainty analysis by comparing flatness, continuity, and resolution of reflections in KPSDM common image gathers. Analysis for location S4 as indicated by the label and vertical dashed line in Fig. S1. The green arrow points to the reflection from top of oceanic crust.

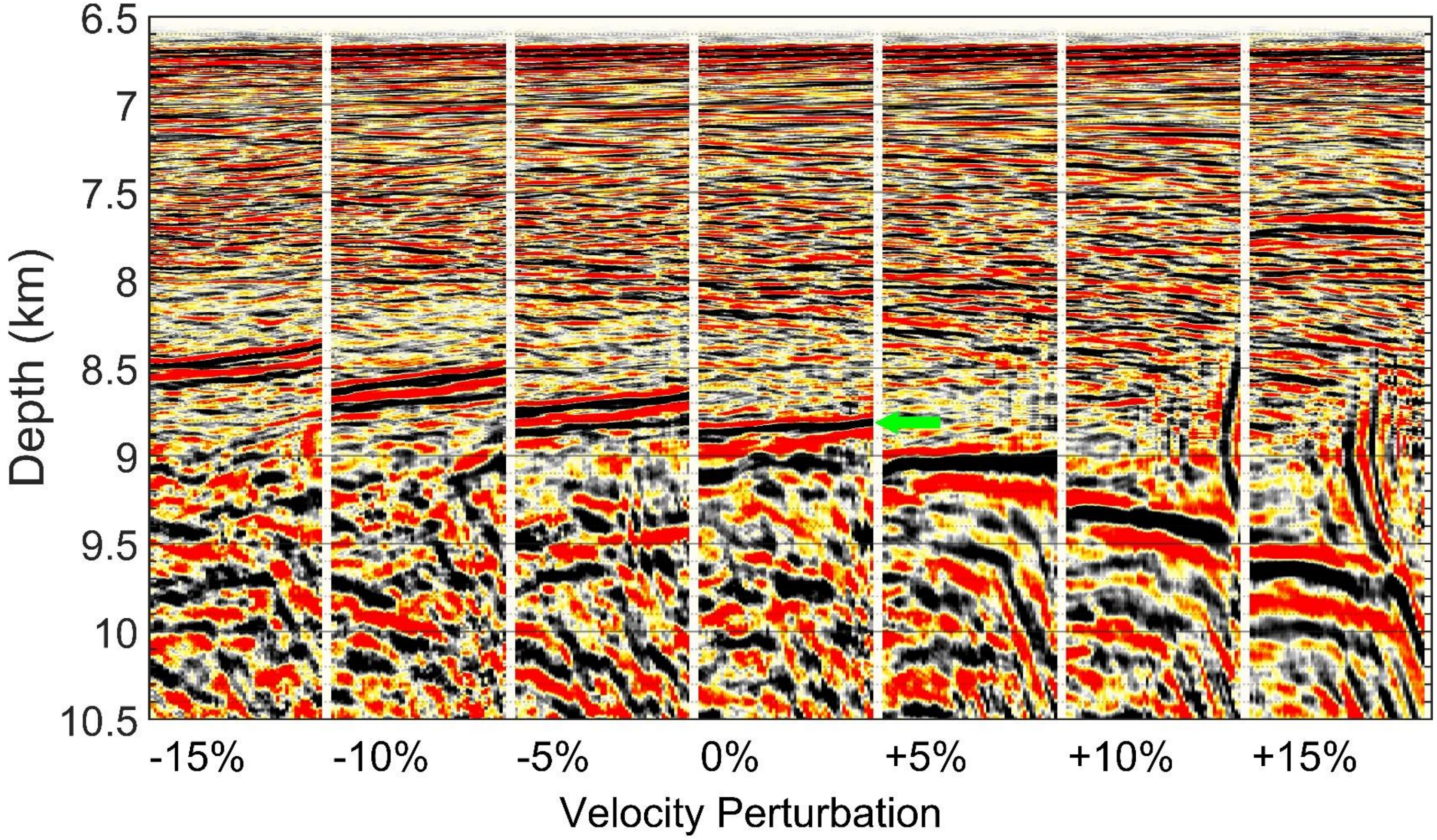

Fig. S5 | Velocity uncertainty analysis by comparing flatness, continuity, and resolution of reflections in KPSDM common image gathers. Analysis for location S5 as indicated by the label and vertical dashed line in Fig. S1. The green arrow points to the reflection from top of oceanic crust.

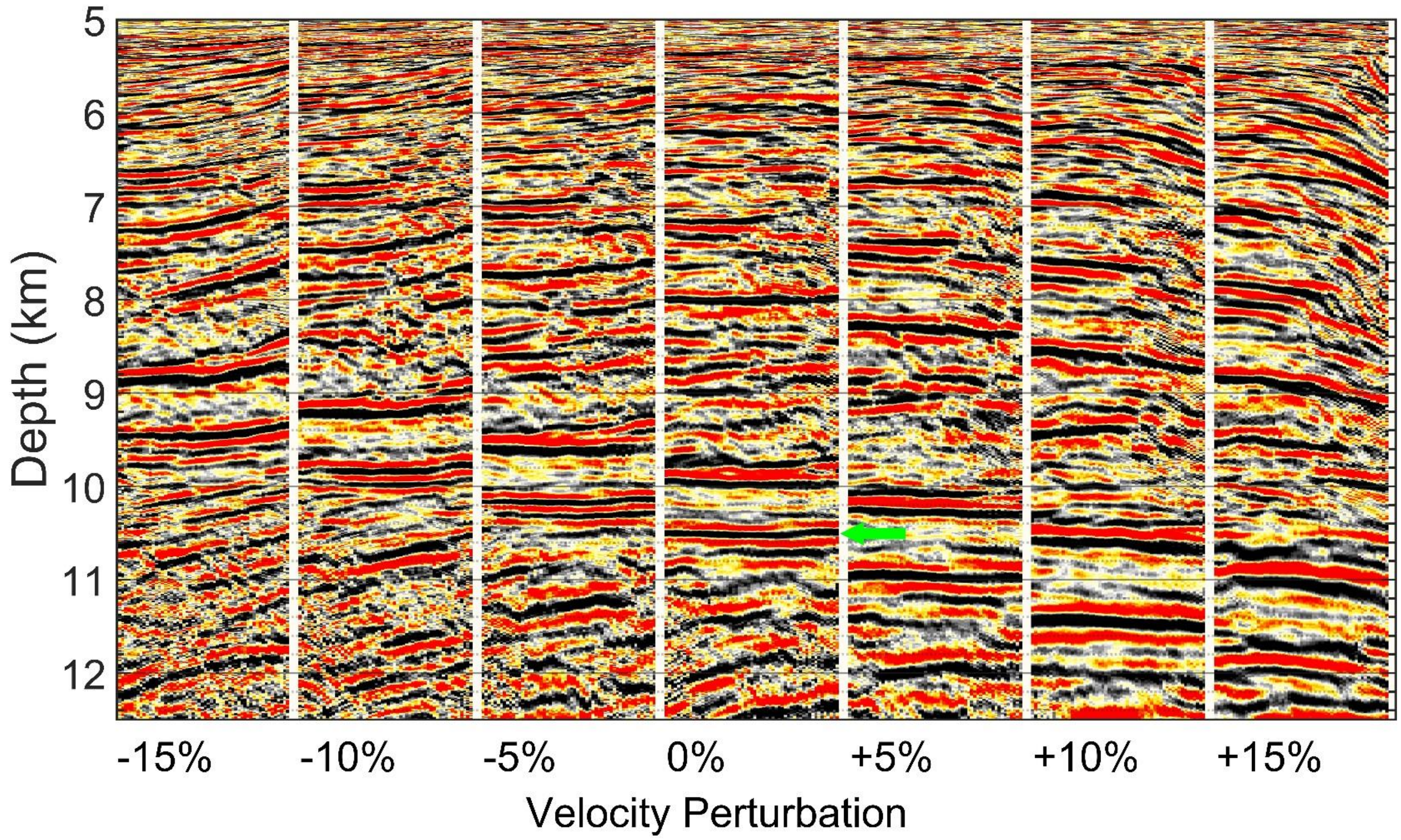

Fig. S6 | Velocity uncertainty analysis by comparing flatness, continuity, and resolution of reflections in KPSDM common image gathers. Analysis for location S6 as indicated by the label and vertical dashed line in Fig. S1. The green arrow points to the reflection from top of oceanic crust.

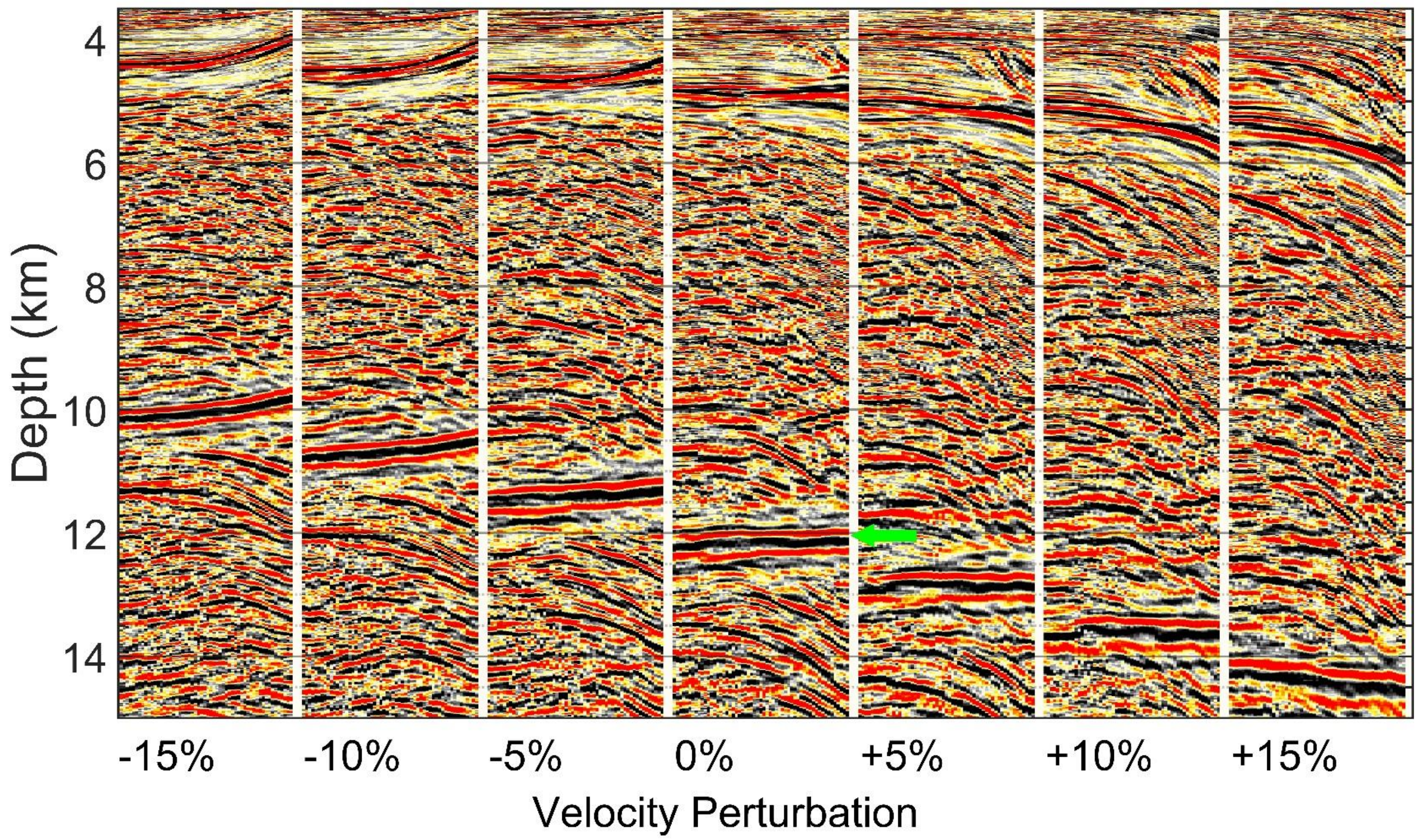

Fig. S7 | Velocity uncertainty analysis by comparing flatness, continuity, and resolution of reflections in KPSDM common image gathers. Analysis for location S7 as indicated by the label and vertical dashed line in Fig. S1. The green arrow points to the reflection from top of oceanic crust.

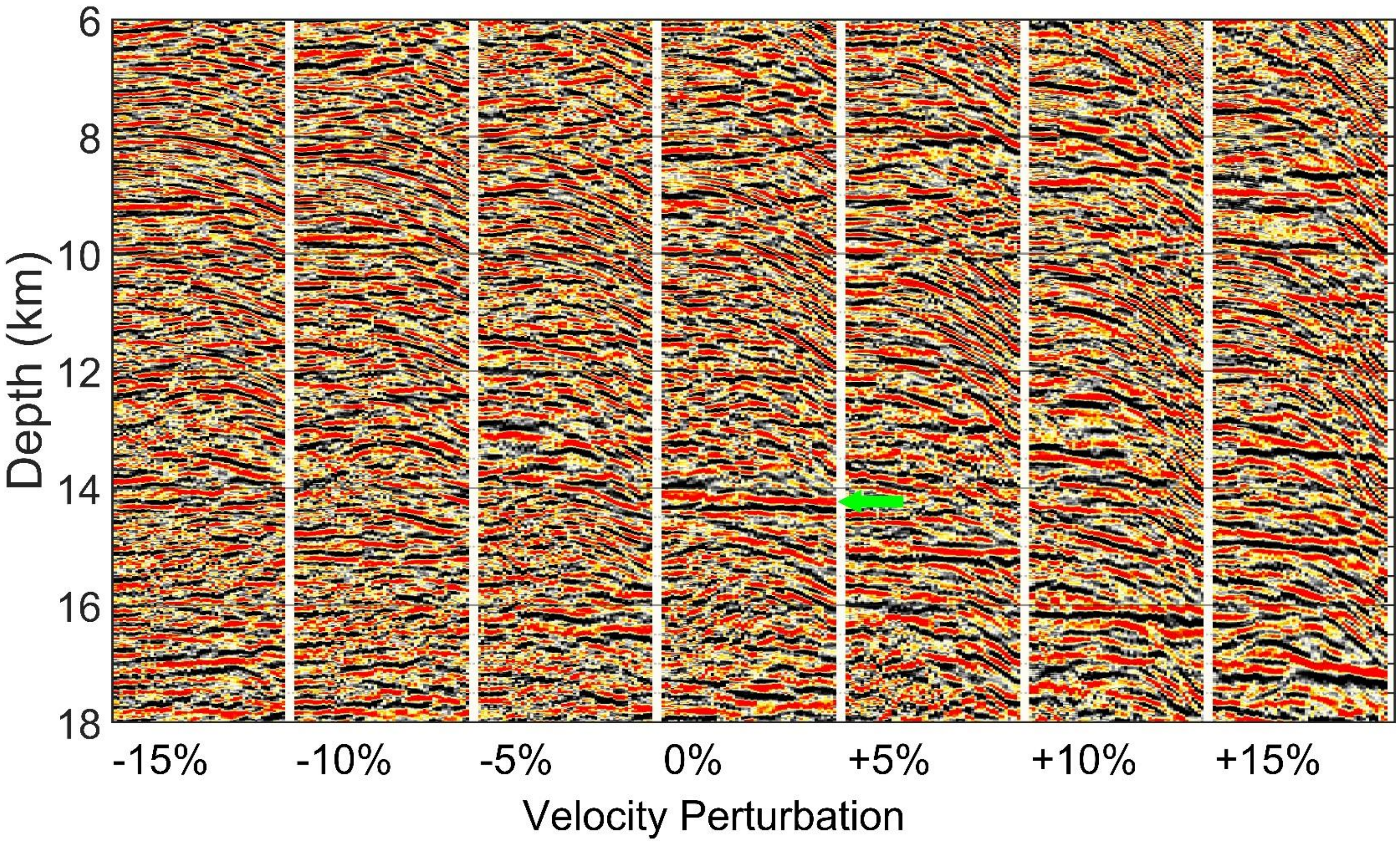

Fig. S8 | Velocity uncertainty analysis by comparing flatness, continuity, and resolution of reflections in KPSDM common image gathers. Analysis for location S8 as indicated by the label and vertical dashed line in Fig. S1. The green arrow points to the reflection from top of oceanic crust.

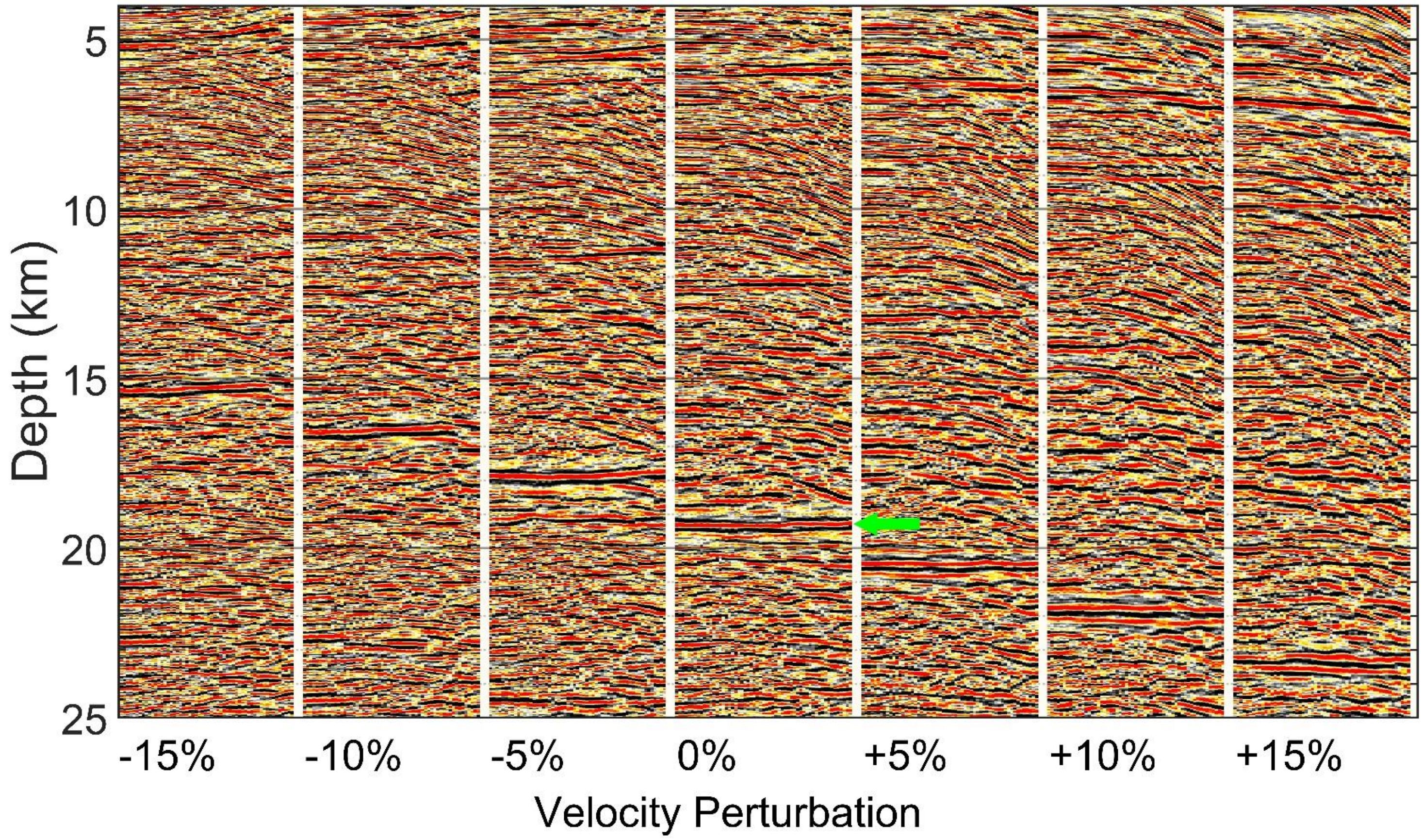

Fig. S9 | Velocity uncertainty analysis by comparing flatness, continuity, and resolution of reflections in KPSDM common image gathers. Analysis for location S9 as indicated by the label and vertical dashed line in Fig. S1. The green arrow points to the reflection from top of oceanic crust.

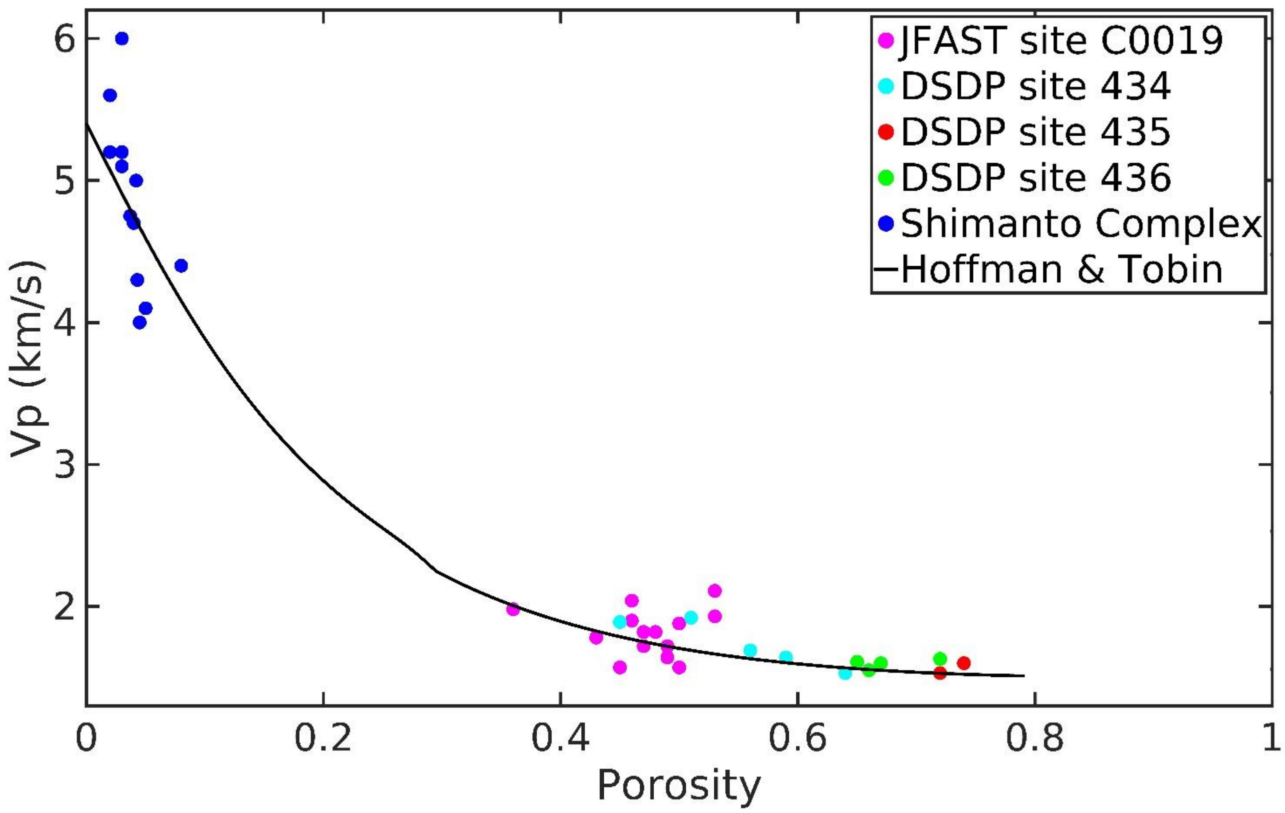

Fig. S10 | P-wave velocity versus porosity. Plotted for the core samples retrieved from IODP JFAST site C0019, DSDP sites 434, 435, 436, and Shimanto complex laboratory experiments[2]. The empirical relationship[1] is in good agreement with the core samples and can be used in Japan Trench.

(a)

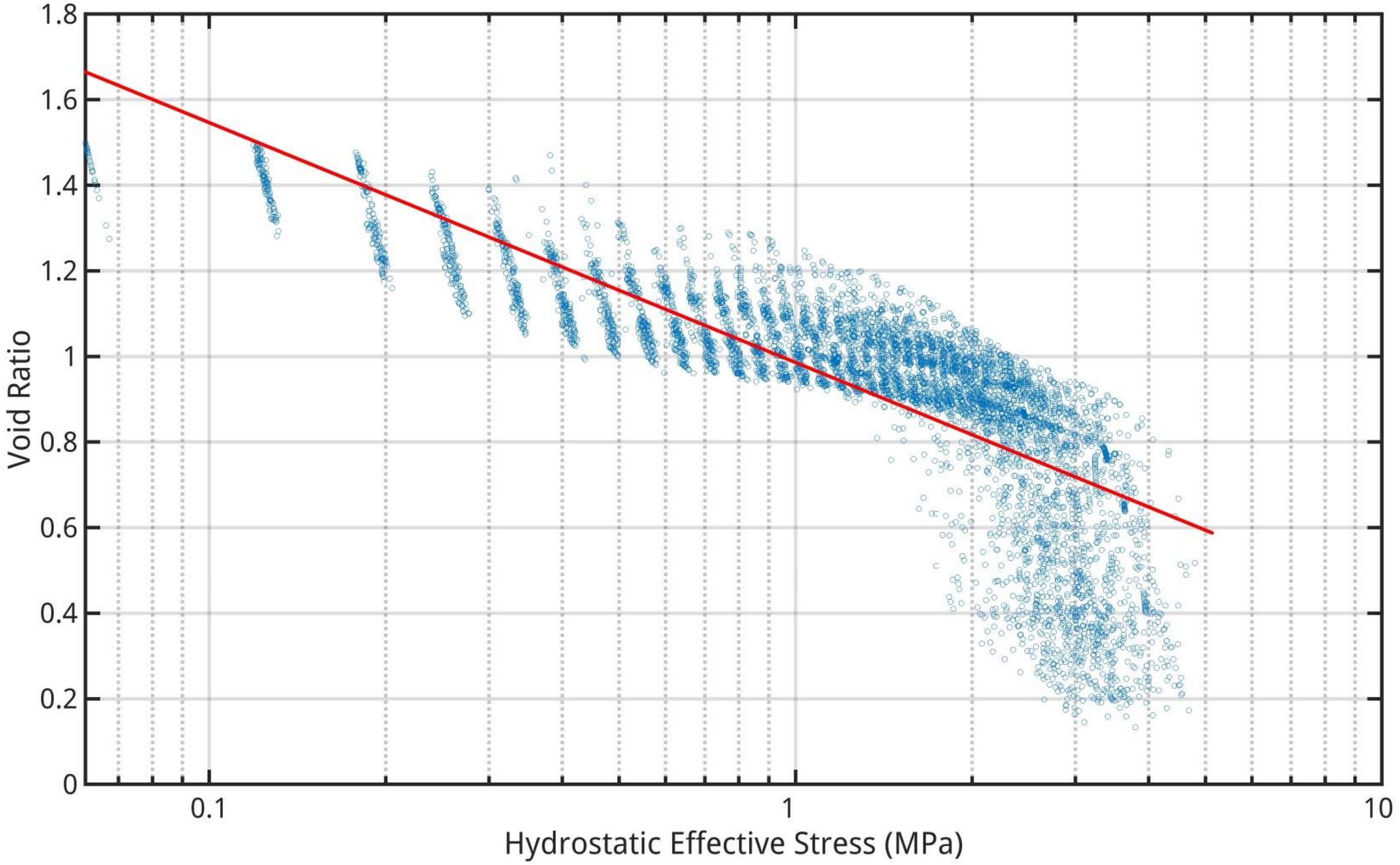

1.8
1.6
1.4
1.2
1
0.8
0.6
0.4
0.2
0
Void Ratio
0.1
1
10
Hydrostatic Effective Stress (MPa)

(b)

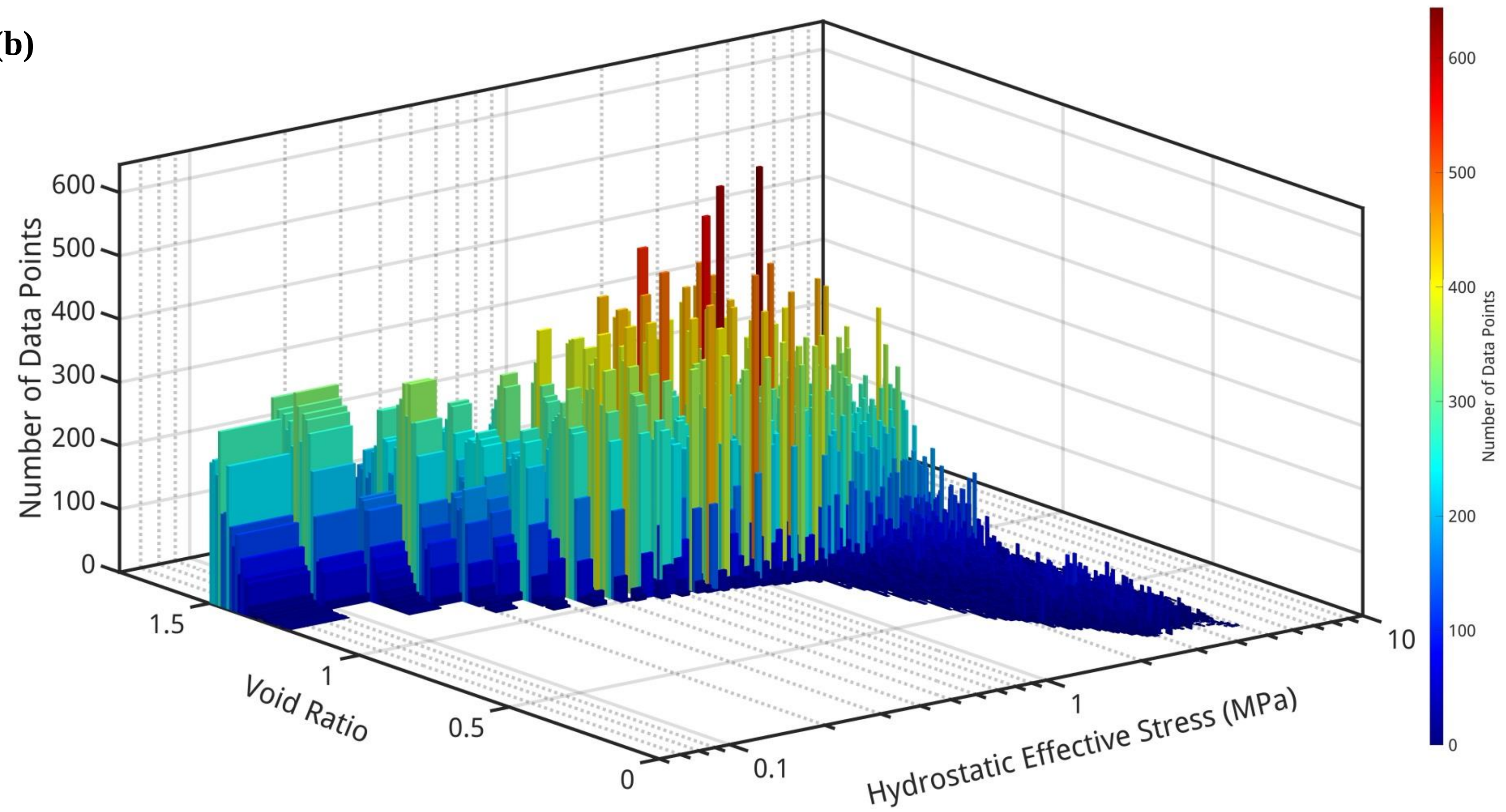

Number of Data Points
600
500
400
300
200
100
0
1.5
1
0.5
0
Void Ratio
0.1
1
10
Hydrostatic Effective Stress (MPa)
Number of Data Points
600
500
400
300
200
100
0

(c)

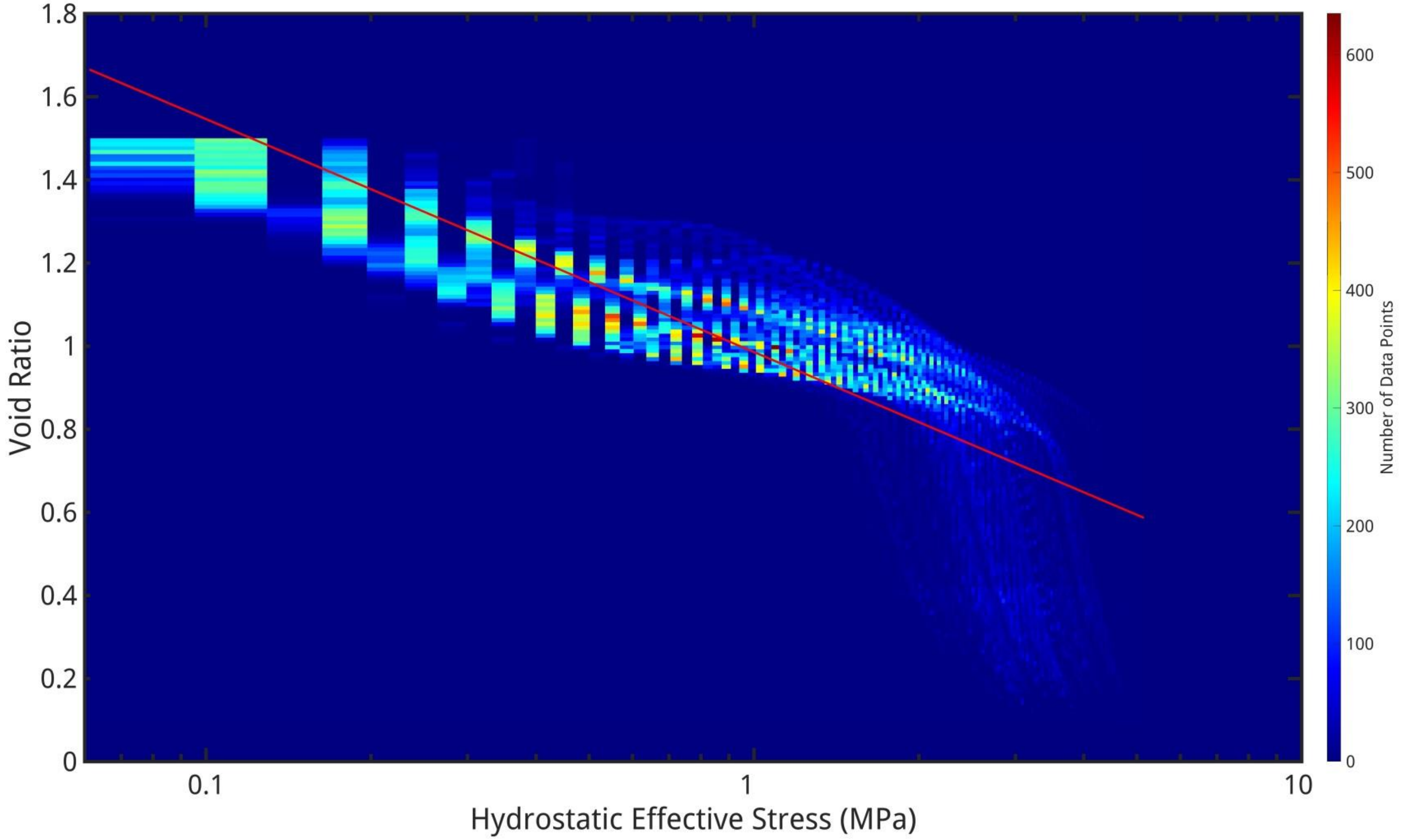

1.8
1.6
1.4
1.2
1
0.8
0.6
0.4
0.2
0
Void Ratio
0.1
1
10
Hydrostatic Effective Stress (MPa)
600
500
400
300
200
100
0
Number of Data Points

Fig. S11 | Logarithmic-linear consolidation curve fitting. (a) The vertical effective stress for the undeformed incoming sediments is calculated from the beginning of the seismic profile (at -50 km horizontal distance) toward the trench, where pore fluid pressure is assumed to be hydrostatic (blue circles). Then a logarithmic-linear relationship is fitted (red line) between void ratio and hydrostatic effective stress to build the consolidation curve for extracting vertical effective stress of the underthrust sediments. For the sake of visual clarity, only 2% of the data points which were used for curve fitting are shown here (every 50th point). **(b)** A 3D histogram showing the distribution of the whole data points, where the height of the columns and the color bar show how densely the data points are located. **(c)** A planar top-view of the 3D histogram which shows that the fitted logarithmic-linear function passes through the highly dense data points.
